# Populism, Exclusion, Post-truth. Some Conceptual Caveats: Comment on "The Rise of Post-truth Populism in Pluralist Liberal Democracies: Challenges for Health Policy"

**DOI:** 10.15171/ijhpm.2017.80

**Published:** 2017-07-15

**Authors:** Benjamin De Cleen

**Affiliations:** ^1^Department of Communication Studies, Vrije Universiteit Brussel-VUB, Brussels, Belgium.; ^2^Center for Media Data and Society, and Political Science Department, Central European University, Budapest, Hungary.

**Keywords:** Nationalism, Populism, Post-Truth

## Abstract

In their editorial, Speed and Mannion identify two main challenges "the rise of post-truth populism" poses for health policy: the populist threat to inclusive healthcare policies, and the populist threat to well-designed health policies that draw on professional expertise and research evidence. This short comment suggests some conceptual clarifications that might help in thinking through more profoundly these two important issues. It argues that we should approach right-wing populism as a combination of a populist down/up (people/elite) axis with an exclusionary nationalist in/out (member/non-member) axis. And it raises some questions regarding the equation between populism, demagogy and the rejection of expertise and scientific knowledge.

Populism has been a hot topic for many years now. It has served as one of the main interpretive frameworks for understanding a wide range of political phenomena: European populist radical right parties like the French Front National, Venezuela’s Hugo Chávez and other left-wing leaders in Latin America, European left-wing anti-austerity movements such as the Greek SYRIZA and the Spanish PODEMOS, the Brexit campaign in the United Kingdom, and most recently, Donald Trump’s rise to power. Whilst of earlier coinage and certainly not denoting an entirely new phenomenon, the term ‘post-truth politics’ has gained prominence more recently in the context of Trump’s election campaign and presidential communication strategies.

Speed and Mannion’s editorial reflects on the challenges of “the rise of post-truth populism” for health policy.^1^ Two such challenges are central to their argument: the populist threat to inclusive and accessible healthcare policies, and the populist threat to well-designed health policies that draw on professional expertise and research evidence. These are matters of major significance indeed. I certainly do not have the expertise to evaluate the impact of populist politics on health policy, and I can only agree with Speed and Mannion’s warnings about the dangers for health policies posed by populist politicians such as Donald Trump or right-wing Brexiteers. My aim in this piece is to suggest some conceptual clarifications that, I hope, might help in thinking through more profoundly these two important questions about the relations between populist politics and health policy.

## Populism, Nationalism, Exclusion

Populism, Speed and Mannion argue, undermines the principle of universal healthcare. From Trump’s anti-Mexican rhetoric to the rejection of Easter-European immigrants and Syrian refugees in the Brexit debate, they write, populism “discriminates against certain sub-sections of the population and exacerbates existing national (and global) health inequalities.” Moreover, they argue, populists’ nationalist protectionism threatens the benefits for healthcare of “international cooperation and agreements that allow the free flow of people, capital, goods and information.”

These arguments echo a more general and very common view of populism as inherently opposed to national outgroups and globalisation. In fact, Speed and Mannion’s theorization of populism does not make the mistake of conflating populism with xenophobia or the radical right, characteristic of European analyses of populism especially.^2^ Drawing on the work of Laclau, Weyland, and Mudde, they stress that populist politics revolves around the antagonism between ‘the people’ and ‘the elite.’ “Populism,” they write, “concerns only the antagonistic relationship between the people and the elite, who is considered to be the elite or the people, depends on the political orientation of the populist.” Populism, therefore, is “neither ostensibly of the right, middle or the left.” Drawing on the conceptualization of populism as a ‘thin ideology,’ mainly associated with the work of Cas Mudde,^3^ they argue that “this ideology can be appended to a range of “thick” ideologies with more mature political logics such as socialism and nationalism.”

However, as a consequence of their focus on right-wing populists such as Trump and the UK Independence Party they then go on to treat populism as inherently nationalist, xenophobic or even racist. For example, they argue that “[p]opulism […] is concerned with national protectionism which limits international cooperation and movement,” which the authors consider to threaten advancements in healthcare. Later on they write that “[t]here are clear parallels with the events in Europe in the 1930s, with populist claims of putting the people first, while promoting division and turning people against one another.”

Right-wing populists like UKIP, Trump or the Front National are most certainly nationalist and exclusionary. However, these are not an inherent feature of populism, as Speed and Mannion themselves indicate in their conceptualization of populism. A more consistent focus on how populism, is *combined* with different ideologies, and used to further those ideologies, allows us to better grasp the potential threats populism poses to a broadly accessible healthcare system. For one, the welfare chauvinism of populist (radical) right politics that undermines universal access to healthcare and other welfare provisions, is structured by the ethnic-cultural in/out (member/non-member) nativist dimension of their politics, not by the populist down/up (people/elite) dimension in itself.^4,5^ And the backlash against birth control services or forms of sexual orientation discrimination Speed and Mannion mention, are not inherent to populism either, but to the programmes of the (radical) right. If we aim to grasp the challenges (radical) right-wing populist politics pose for health policy we need to think carefully about which dimensions of these politics threaten universal and accessible healthcare provisions and in what ways. Populist strategies have certainly contributed significantly to strengthening the appeal of nativist and radical right politics beyond a core of traditional radical right-wing activists and voters, but the threat posed by such parties cannot be grasped through the term populism alone. Using ‘populism’ as shorthand for these kinds of politics might actually shift our attention away from what *really* matters to these parties (exclusionary nationalism) and from how they *really* threaten solidarity and democracy (by excluding refugees, migrants, people of foreign descent). Instead, we need to ask how the populist down/up (people/elite) axis and the nationalist in/out (member/non-member) axis are articulated in right-wing exclusionary populisms, and combined with other elements of radical right-wing programmes.

Secondly, populist politics are not necessarily nationalist, and certainly not necessarily xenophobic. Bolivia’s Evo Morales in Bolivia, US Presidential candidate Bernie Sanders, or Spain’s PODEMOS are certainly populist. But if anything their politics have been about the inclusion of marginalised ethnic groups, rather than their exclusion. Moreover, in this kind of left-wing populisms, the populist strategy of claiming to represent ‘the people’ against ‘the elite’ is used to formulate demands for solidarity, for more equitable and universal access to welfare provisions. This happens through the formulation of a broader and more exclusive definition of ‘the people,’ that unlike on the right, is not limited to the nation, defined in an exclusionary ethnic-cultural way.^6^ Such left-wing populist movements, therefore, can use populism to demand and implement, in the name of ‘the people,’ more inclusive and accessible health care provisions; think for example about the significant investments in health care in Venezuela under Hugo Chávez or about Bolivia’s Evo Morales’ promises of free healthcare for all. Left-wing populists can formulate their criticism of neoliberal privatisation of or austerity measures in healthcare in populists terms, accusing the ‘the elite’ of betraying ‘the people’ and serving its own interest by privatising health care provision (the NHS, for example), whilst demanding the (re)nationalisation of healthcare in the name of ‘the people.’

The relation between populism and inclusion and exclusion from healthcare and other welfare provisions is therefore less straightforward than the almost completely negative picture Speed and Mannion draw as a consequence of their empirical focus on Trump and Brexit. As Speed and Mannion’s own theoretical framework suggests, we can only grasp the potential threats and potentials of populist politics for health policy by looking at the interconnection of populism with other components of these politics, “such as socialism and nationalism.”

## Populism, Expertise and “Post-truth”

A second challenge of ‘post-truth populism’ for health policy, according to Speed and Mannion, lies in populism’s rejection of expertise and scientific knowledge. Populism, they write, threatens “good [health] policy design” because “populist policies tend to be shaped more by the personal whims and prejudices of a demagogue than underpinned by a secure evidence base.” Moreover, populists also practice ‘post-truth’ politics to attract voters: “Populist politicians’ reliance on assertions that appear true, but have no basis in fact, creates a false view of the world, not with the intention of convincing the elites that they are right, but in reinforcing prejudices among their targeted pool of potential supporters.”

Whereas the inclusionary or exclusionary nature of different kinds of populist politics depends almost entirely on components other than populism, the down/up, people versus elite antagonism inherent to populism does indeed lend itself very well to a celebration of ordinary people’s opinions. And it is easily used to delegitimize experts as members of an ‘elite’ that is out of touch with ordinary people and that does not serve their interests.

I would like to suggest some conceptual precisions that might help us to analyse and evaluate populists’ relationship with expertise, knowledge, factuality and demagogy more thoroughly. It seems useful here to distinguish between the use of expertise in policy design, and expert-led technocratic decision-making; that is, between the use of expertise in designing policies that pursue aims decided on through democratic procedures, and experts setting policy priorities.

On the first level, the questions are: Do populist politicians use expertise to formulate their policy proposals? Do they, once in power, draw on scientific experts and professional civil service to achieve the policy goals they have formulated? Or is there something inherently anti-expertise in populist politics? How we define populism is important here. If we define populism as a thin ideology that revolves around the belief in a ‘pure people’ and ‘a corrupt elite’ and the formulation of policies based on that belief,^7^ then populism would indeed tend to anti-expert positions. However, if we, as I would prefer, stress populism’s strategic rather than ideological nature and see populism as a particular political logic – a particular way of formulating demands as based in ‘the people,’ of interpellating citizens as members of the people-as-underdog, and of criticising opponents as an illegitimate ‘elite’^8,9^– then populism does not necessarily entail distrust for expert knowledge *per se*. Not unlike other politicians, populists are likely to draw on the expertise that suits their aims and to disregard expertise that does not. At the same time, the populist logic does revolve around the celebration of ‘the people,’ and it is particularly suitable to the anti-intellectualist delegitimation of expertise that does not fit a particular populist party’s goals as ‘elitist’ and ‘far removed from the people.’ Whilst not a necessary characteristic of populism, it is certainly worth researching whether “populist policies tend to be shaped more by the personal whims and prejudices of a demagogue than underpinned by a secure evidence base,” as Speed and Mannion suggest. If this is the case, this definitely holds risks for well-designed health policy as “the disdain for policy experts by politicians pursuing populist policies, may result in poorly designed and implemented health policies with potentially seriously dysfunctional consequences.”

The latter argument can also be read on a second level, however, where the populist “disdain for policy experts” starts to mean something else, and where the critique of populism itself risks to become democratically problematic. A second level of the analysis between populists and experts might indeed revolve around the question: Who should determine policy goals and on what basis? Should experts determine policy goals or should the electorate? And if the latter, how directly and through what channels? And also: Should the electorate base its political choices on expertise? Populist politicians have criticised technocratic decision-making as undemocratic for not taking into account the opinions of the people. Populist politics, as Speed and Mannion also write, have been described by many as a correction to and consequence of the move of decision-making power away from ordinary citizens and directly elected representatives to supra-national bodies, technocrats, as well as away from democratic politics to the private sector.^10-12^ This diagnosis of populism links up with broader criticisms of what has been called our ‘post-democratic’^13^ or ‘post-political’ situation. Political theorists such as Chantal Mouffe^14,15^ and Jacques Rancière^16^ have formulated critiques of technocratic and consensual politics that, they argue, deny the properly ‘political’ moment of decision-making: the moment of struggle between differing opinions and interests. By presenting what are fundamentally *political* decisions as mere technical *policy* issues, Mouffe and others argue, technocratic ‘center’ politics hide their own political nature behind a veil of objectivity, neutrality, and value-free expertise. Simultaneously, these supposedly value-free and rational decisions are opposed to the supposedly irrational and emotional whims of the people, and to the populist politics that supposedly play to people’s prejudices and ignorance. This is where the longstanding dismissal of populist politics as playing to the underbellies of ordinary people (rather than their brains) and the recently more prominent critique of ‘post-truth’ come together.

Post-truth, Speed and Mannion cite the Oxford dictionary “relates to or denotes circumstances in which objective facts are less influential in shaping public opinion than appeals to emotion and personal belief.” Certain kinds of populist politics exploit and strengthen prejudice and ignorance among parts of the population, and some of them do through lies and deceitful propaganda (Trump and parts of the Brexit campaign are good examples indeed). We should be careful not to equate populism with demagogy or ‘post-truth politics,’ however. The populist pitting of the people against the elite is not necessarily demagogic or dishonest (and can even be used to oppose demagogic and dishonest politics). Moreover, the critique of ‘post-truth populist politics’ can lead us to a problematic delegitimization of ‘the people’ as led by emotions rather than well-informed opinions. The idea that “objective facts” (should) shape public opinion – implied in the Oxford definition of post-truth for example – also loses sight of the unavoidable emotional and affective elements of all kinds of politics, from Left to Right, from inclusionary to exclusionary, from communist to neoliberal, and from populist to anti-populist. Furthermore, we should be careful not to treat the knowledge produced by experts as value-free or neutral (as opposed to non-neutral political views). Indeed, expert knowledge can, and has, been used to strengthen the welfare state *as well as* to undermine its legitimacy and replace the institutions of the welfare state with profit-driven market structures.

## Conclusion

It looks as if populist political parties and leaders are not likely to retreat from the front stage anytime soon. The concept of populism is absolutely crucial in furthering our understanding of the political strategies and popular appeal of populists on the Left and the Right, from PODEMOS to the Front National and from Sanders to Trump. We need to continue to investigate the threats populist politics pose to democratic politics and just societies, as Speed and Mannion argue. But we also need to consider the potentials they might offer for a more democratic and more inclusive politics. In drawing on a conceptualization of populism as revolving around a people versus elite antagonism, Speed and Mannion’s piece starts from a sound basis for the analysis of the threats and potentials of populist politics for an equitable health policy, and points out a number of crucial challenges of right-wing exclusionary ‘post-truth’ populism poses to health policy. Such an analysis would also benefit, I have suggested in this text, from a more rigorous restriction of the concept of populism to that people/elite dimension.

The term populism has great analytical potential, but it never suffices to cover the entire politics of any populist party, leader or movement. To grasp how populist politics of the nationalist and xenophobic kind might impact on health policy, we need to look at the articulation of populism with nationalism and xenophobia, and at how populist strategies legitimise nationalist and xenophobic exclusion. Similarly, to probe the consequences of ‘post-truth populism’ for health policy, we need to ask how certain populists connect the people-elite antagonism with a ‘post-truth’ disregard of factuality and a delegitimation of expertise. I hope the conceptual clarifications I have suggested in this brief contribution might prove of use in in thinking through the issues of major societal importance raised by Speed and Mannion.

## Ethical issues

Not applicable.

## Competing interests

Author declares that he has no competing interests.

## Author’s contribution

BDC is the single author of the paper.
